# The Synthesis Model of Flat-Electrode Hemispherical Resonator Gyro

**DOI:** 10.3390/s19071690

**Published:** 2019-04-09

**Authors:** Zhennan Wei, Guoxing Yi, Yan Huo, Ziyang Qi, Zeyuan Xu

**Affiliations:** Space Control and Inertial Technology Research Center, Harbin Institute of Technology, Harbin 150080, Heilongjiang, China; wzn@hit.edu.cn (Z.W.); hy_hit@163.com (Y.H.); qi.ziyang@protonmail.com (Z.Q.); xuzeyuan@hit.edu.cn (Z.X.)

**Keywords:** hemispherical resonator gyro, flat electrode, motion equation, excitation and detection model, assemble error and parameter, identification method

## Abstract

The Hemispherical Resonator Gyro (HRG) is a solid-state and widely used vibrating gyroscope, especially in the field of deep space exploration. The flat-electrode HRG is a new promising type of gyroscope with simpler structure that is easier to be fabricated. In this paper, to cover the shortage of a classical generalized Coriolis Vibration Gyroscope model whose parameters are hard to obtain, the model of flat-electrode HRG is established by the equivalent mechanical model, the motion equations of unideal hemispherical shell resonator are deduced, and the calculation results of parameters in the equations are verified to be reliable and believable by comparing with finite element simulation and the reported experimental data. In order to more truthfully reveal the input and output characteristics of HRG, the excitation and detection models with assemble errors and parameters are established based on the model of flat-electrode capacitor, and they convert both the input and output forms of the HRG model to voltage changes across the electrodes rather than changes in force and capacitance. An identification method of assemble errors and parameters is proposed to evaluate and improve the HRG manufacturing technology and adjust the performance of HRG. The average gap could be identified with the average capacitance of all excitation and detection capacitors; fitting the approximate static capacitor model could identify the inclination angle and direction angle. With the obtained model, a firm and tight connection between the real HRG system and theoretical model is established, which makes it possible to build a fully functional simulation model to study the control and detection methods of standing wave on hemispherical shell resonator.

## 1. Introduction

The Hemispherical Resonator Gyro (HRG) is a solid-state vibrating gyroscope that is based on inertial effect of elastic wave and it is well-known for its excellent feature, such as high precision, energy efficiency, long working life, and extremely simple structure. In the efforts of Northrop Grumman Corporation and Safran Electronics & Defense, the HRG is used widely at present, including land, ocean, air, and space areas [1,2,3]. Especially in the field of deep space exploration, HRG has been involved in a large number of planet and asteroid exploration missions due to its legendary reliability and stationarity. On October 15, 1997, the Cassini-Huygens probe that is equipped with HRG was launched from Cape Canaveral Air Force Station’s Space Launch Complex 40, and it was active in space for nearly 20 years until the end of the mission. The navigation data of Cassini during nearly 20 years proves the excellent performance of HRG in space applications [4,5].

For the purpose of mass production, Sagem, which started to develop HRG in 1985, successfully fabricates a new type HRG, which is a so-called flat-electrode HRG and it consisted of only six parts [6,7,8,9]. When compared with the three-dimensional electrode HRG applied in Cassini mission [10,11,12], the structure of flat-electrode HRG is simpler and more compact, which leads to the wide application of flat-electrode HRG [13], and it is more promising.

At present, in the field of HRG theory research, generous theoretical achievements have been obtained for the model of HRG, but some issues, especially in the combination of theoretical model with practical hardware, are still unresolved.

In reference [14], generalized Coriolis Vibration Gyroscope (CVG) motion equations are provided, in which the misalignment of the inherent frequency axis and the inherent damping axis is taken into account, and they are the most widely used motion equations, which are even included in the IEEE Standards [15]. However, the obtaining method of coefficients in the equations for HRG is not given, and the excitation and detection models of standing wave are also unknown. Reference [16] provides the motion equations of Coriolis Vibrating Gyroscope in an ideal state, and the analysis of excitation and detection methods on mathematics, but still no obtaining method of coefficients in the equations for HRG. In reference [17], the motion equations of the spherical shell are deduced by using Lagrange Equation, and the free vibration of shell is discussed in the absence of excitation and detection models. In reference [18], the hemispherical shell is equivalent to a particle, a two-dimensional (2-D) damping-spring equivalent vibration model is established, and the methods of controlling and detecting standing wave is discussed, but the method calculating the value of equivalent mass is not stated. In reference [19], the hemispherical shell is equivalent to a ring, the effects of assemble parameters, physical dimension, and Q-factor on resonator motion model are only qualitatively analyzed. In reference [20], the motion equations of HRG are established based on the Bubnov–Galerkin method, but the inherent frequency of resonator deduced by the equations is inaccurate, and it is far away from experimental results [21] or finite element simulation results [22,23], which indicates the inaccuracy of the equations. In reference [24,25], the free motion equations of ideal hemispherical shell are deduced by using the Lagrange Equation, but the error and control analyses still rely on the equations that are provided by reference [14], and the misalignment parameters between axes are misunderstood. Reference [26] presents a fully functional HRG simulation system based on the equations that are provided by reference [14] and the excitation and detection models omitting the distance deviation due to the assemble error of resonator. 

In summary, within the theoretical research fields of HRG, especially for flat-electrode HRG, some vital motion equations describing the HRG vibration performance under the condition of misalignment between forcer-pickoff axes, inherent frequency axes, and inherent damping axes are still missing when the external control forces act. At the same time, the excitation and detection models of HRG, which describe the methods of applying electrostatic force through capacitors and the effects of vibration displacement on capacitance of capacitors, are not complete. 

In this paper, these problems have been solved. The motion equations of flat-electrode HRG containing misalignment between the inherent characteristic axes have been deduced, and the excitation and detection models with assemble errors have been established. With these equations, a tight connection between the real HRG system and theoretical model is established, and varying voltage signals applied across exciting electrodes can lead to varying voltage signals across detecting electrodes. Besides, an identification method of assemble errors and parameters that are involved in excitation and detection models is proposed, which will be beneficial to the gain compensation of electrodes and it could evaluate and improve the manufacturing technology of HRG. Finally, a fully functional simulation model can be established based on the equations obtained, and the control and detection methods of standing wave of HRG can be studied with these equations in the future. 

## 2. Motion Equations of Hemispherical Shell Resonator

In this section, the motion equations of hemispherical shell resonator with several error sources and control forces have been deduced. Firstly, by applying the Lagrangian Mechanics Principle and the Elastic Shell Theory, the motion equations of ideal hemispherical shell with perfect dimension and material parameters are deduced. Secondly, the direction angles of inherent frequency axes and inherent damping axes are involved into the ideal motion equations by coordinate transformation. Finally, multi-directional driving forces and inertia force have also been introduced in the motion equations by the vector composition method, and the motion equations of hemispherical resonator that are suitable for full functional response simulation of HRG are obtained. Not only are the final motion equations suitable for analysis of flat-electrode HRG, as mentioned in later sections, but also the three-dimensional electrode HRG.

### 2.1. Motion Equations of Ideal Hemispherical Shell Resonator

**Figure 1:**
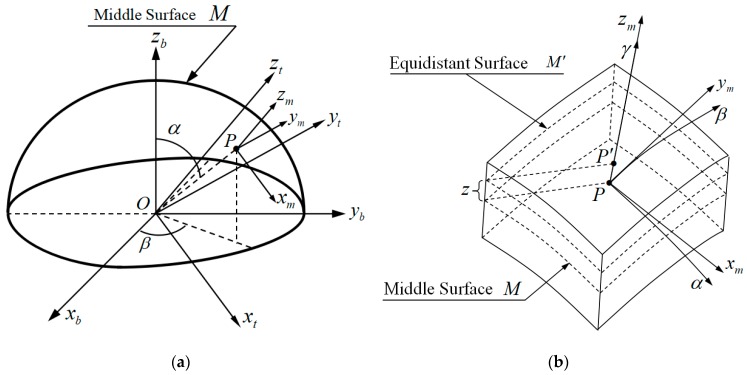
Middle surface, equidistant surface and corresponding coordinate systems: (**a**) Middle surfaceof hemispherical shell and corresponding coordinate systems; and, (**b**) Equidistant Surface $M^{\prime }$ of hemispherical shell and corresponding coordinate systems.

Figure 1a shows the middle surface M of hemispherical shell and the corresponding coordinate systems. Equidistant Surface $M^{\prime }$ of hemispherical shell and the corresponding coordinate systems are shown in Figure 1b. There are four coordinate systems in Figure 1, namely:

1. Orthogonal Curvilinear Coordinate System P−αβγ: Origin P is located on M, axis γ is perpendicular to M, axes α, β lie in M, and axes α, β, γ are orthogonal to each other.
2. Middle Surface Coordinate System $P-x_{m}y_{m}z_{m}$: Origin P is located on M, axes $x_{m}$, $y_{m}$, $z_{m}$ are parallel to the tangential directions of axes α, β, γ, respectively.
3. Shell Coordinate System $O-x_{b}y_{b}z_{b}$: Origin P is located at the centre of the circular opening of shell, axis $z_{b}$ is coincident with the shell symmetry axis, axes $x_{b}$, $y_{b}$ lie in the circular opening mentioned above, and axes $x_{b}$, $y_{b}$, $z_{b}$ are orthogonal to each other and fixed with the shell.
4. Rotation Coordinate System $O-x_{r}y_{r}z_{r}$: Origin *P* is located at the centre of the circular opening of shell, axes $x_{r}$, $y_{r}$, $z_{r}$ are parallel to axes $x_{m}$, $y_{m}$, $z_{m}$.

According to the Elastic Shell Theory, strains of point $P^{\prime }$ in $M^{\prime }$ of the hemispherical shell could be expanded as a polynomial form of z, and it is described as the following equations [27] (pp. 240–253):

(1) $$\{\begin{array}{l} \varepsilon_{\alpha }^{z}=\varepsilon_{\alpha }^{z_{0}}+z\varepsilon_{\alpha }^{z_{1}}+O_{\alpha }^{z}\left(z^{2}\right)\\ \varepsilon_{\beta }^{z}=\varepsilon_{\beta }^{z_{0}}+z\varepsilon_{\beta }^{z_{1}}+O_{\beta }^{z}\left(z^{2}\right)\\ \gamma_{\alpha \beta }^{z}=\gamma_{\alpha \beta }^{z_{0}}+2z\gamma_{\alpha \beta }^{z_{1}}+2O_{\alpha \beta }^{z}\left(z^{2}\right) \end{array}$$

(2) $$\{\begin{array}{ll} \varepsilon_{\alpha }^{z_{0}}=\frac{1}{R}\frac{\partial u}{\partial \alpha }+\frac{w}{R}, & \varepsilon_{\alpha }^{z_{1}}=-\frac{1}{R^{2}}\frac{\partial^{2}w}{\partial \alpha^{2}}-\frac{w}{R^{2}}\\ \varepsilon_{\beta }^{z_{0}}=\frac{1}{R\sin\alpha }\frac{\partial v}{\partial \beta }+\frac{u\cot\alpha }{R}+\frac{w}{R}, & \varepsilon_{\beta }^{z_{1}}=-\frac{1}{R^{2}\sin^{2}\alpha }\frac{\partial^{2}w}{\partial \beta^{2}}-\frac{\cot\alpha }{R^{2}}\frac{\partial w}{\partial \alpha }-\frac{w}{R^{2}}\\ \gamma_{\alpha \beta }^{z_{0}}=\frac{1}{R}\frac{\partial v}{\partial \alpha }+\frac{1}{R\sin\alpha }\frac{\partial u}{\partial \beta }-\frac{v\cot\alpha }{R}, & \gamma_{\alpha \beta }^{z_{1}}=\frac{\cot\alpha }{R^{2}\sin\alpha }\frac{\partial w}{\partial \beta }-\frac{1}{R^{2}\sin\alpha }\frac{\partial^{2}w}{\partial \alpha \partial \beta } \end{array}$$

where: R is the radius of M. u, v, w are the displacements of point P in M along the direction of axes α, β, γ, and z is the distance between M and $M^{\prime }$ along the direction of axis γ. Let the inner surface radius and outer surface radius of hemispherical shell be $R_{1}$ and $R_{2}$, respectively, then

(3) $$2h=R_{2}-R_{1},R=\left(R_{2}+R_{1}\right)/2,z\in \left[-h,h\right]$$

$\varepsilon_{\alpha }^{z_{0}}$, $\varepsilon_{\beta }^{z_{0}}$ are the normal strains in M, and $\gamma_{\alpha \beta }^{z_{0}}$ is the shear strain in M. $\varepsilon_{\alpha }^{z_{1}}$, $\varepsilon_{\beta }^{z_{1}}$ could be regarded as the change of curvature, and $\gamma_{\alpha \beta }^{z_{1}}$ could be regarded as the change of torsion.

If the hypothesis regarding the deformation of hemispherical shell, there is only bending deformation without extension or compression deformation, is adopted, then

(4) $$\varepsilon_{\alpha }^{z_{0}}=\varepsilon_{\beta }^{z_{0}}=\gamma_{\alpha \beta }^{z_{0}}=0$$

Substituting Equation (4) into Equation (2), the following Equation (5) could be deduced.

(5) $$\{\begin{array}{l} \frac{\partial u}{\partial \alpha }+w=0\\ \frac{1}{\sin\alpha }\frac{\partial v}{\partial \beta }+u\cot\alpha +w=0\\ \frac{\partial v}{\partial \alpha }+\frac{1}{\sin\alpha }\frac{\partial u}{\partial \beta }-v\cot\alpha =0 \end{array}$$

**Figure 2:**
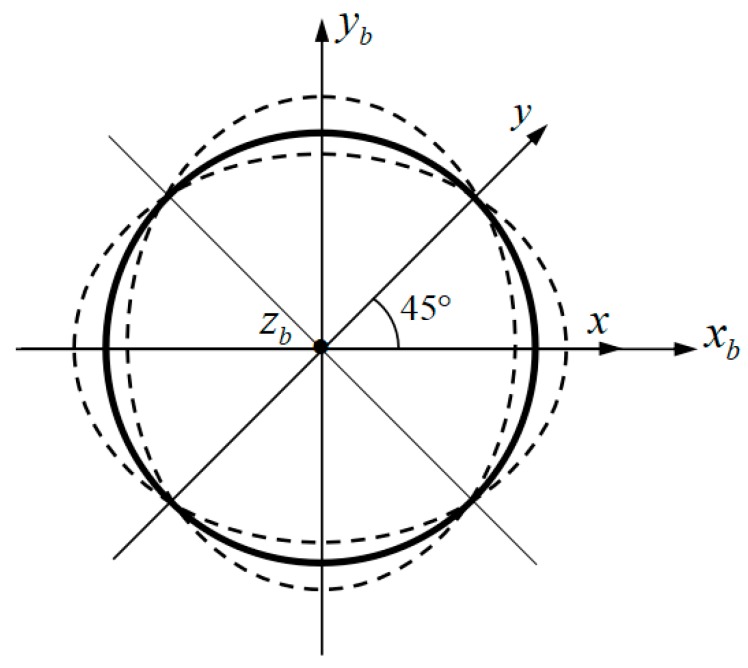
Diagram of second-order vibration mode decomposition.

Assuming that the hemispherical shell vibrates in the second-order mode [28] (p. 156), and displacements of any point in M could be expressed as

(6) $$\{\begin{array}{l} u(\alpha ,\beta ,t)=U(\alpha )\left[x(t)\cos2\beta +y(t)\sin2\beta \right]\\ v(\alpha ,\beta ,t)=V(\alpha )\left[x(t)\sin2\beta -y(t)\cos2\beta \right]\\ w(\alpha ,\beta ,t)=W(\alpha )\left[x(t)\cos2\beta +y(t)\sin2\beta \right] \end{array}$$

where: x(t), y(t) are time-dependent harmonic oscillation functions to reveal the frequency and phase information of hemispherical shell vibration, and Figure 2 shows the directions. U(α), V(α), and W(α) are the gain coefficient of vibration amplitude along axes α, β, γ.

Substituting Equation (6) into Equation (5), the following Equation (7) could be deduced.

(7) $$\{\begin{array}{l} \frac{\partial U}{\partial \alpha }+W=0\\ 2V+U\cos\alpha -\frac{\partial U}{\partial \alpha }\sin\alpha =0\\ \frac{\partial V}{\partial \alpha }\sin\alpha -2U-V\cos\alpha =0 \end{array}$$

The solution of Equation (7) is

(8) $$\{\begin{array}{l} U=V=\sin\alpha \tan^{2}\alpha /2\\ W=-(2+\cos\alpha )\tan^{2}\alpha /2 \end{array}$$

According to the assumption that bending deformation is the only form of hemispherical shell deformation, then the deformation energy [27] (p. 271), potential energy of hemispherical shell, is

(9) $$E_{P}=\frac{E{\left(2h\right)}^{3}}{24(1-\mu^{2})}\int_{0}^{2\pi }\int_{0}^{\frac{\pi }{2}}\left[{\left(\varepsilon_{\alpha }^{z_{1}}\right)}^{2}+{\left(\varepsilon_{\beta }^{z_{1}}\right)}^{2}+2(1-\mu ){\left(\gamma_{\alpha \beta }^{z_{1}}\right)}^{2}+2\mu \varepsilon_{\alpha }^{z_{1}}\varepsilon_{\beta }^{z_{1}}\right]R^{2}\sin\alpha d\alpha d\beta$$

where E and μ are the Yong’s Modulus and Poisson’s Ratio of hemispherical shell material, respectively.

Substituting Equations (2) and (6) into Equation (9), then

(10) $$E_{P}=\frac{1}{2}k_{0}\left(x^{2}+y^{2}\right)$$

where: $k_{0}$ can be regarded as equivalent elastic modulus, and its computing method is

(11) $$k_{0}=\frac{E{\left(2h\right)}^{3}\pi }{12(1-\mu^{2})}\int_{0}^{\frac{\pi }{2}}\left\{\begin{array}{l} \frac{2\mu \sin\alpha }{R^{2}}\left(\frac{\partial W^{2}}{\partial^{2}\alpha }+W\right)\left[\left(1-\frac{4}{\sin^{2}\alpha }\right)W+\frac{\partial W}{\partial \alpha }\cot\alpha \right]+\\ \frac{\sin\alpha }{R^{2}}{\left(\frac{\partial W^{2}}{\partial^{2}\alpha }+W\right)}^{2}+\frac{8(1-\mu )}{R^{2}\sin\alpha }{\left(\frac{\partial W}{\partial \alpha }-W\cot\alpha \right)}^{2}+\\ \frac{\sin\alpha }{R^{2}}{\left[\left(1-\frac{4}{\sin^{2}\alpha }\right)W+\frac{\partial W}{\partial \alpha }\cot\alpha \right]}^{2} \end{array}\right\}d\alpha$$

Rotation matrix from coordinate $O-x_{b}y_{b}z_{b}$ to coordinate $P-x_{m}y_{m}z_{m}$ in Figure 1a is

(12) $$C_{b}^{m}=\left[\begin{array}{ccc} \cos\alpha \cos\beta & \cos\alpha \sin\beta & -\sin\alpha \\ -\sin\beta & \cos\beta & 0\\ \cos\beta \sin\alpha & \sin\alpha \sin\beta & \cos\alpha \end{array}\right]$$

$C_{b}^{m}$ is also the rotation matrix from $O-x_{b}y_{b}z_{b}$ to $O-x_{r}y_{r}z_{r}$ exactly, which means that $C_{b}^{m}=C_{b}^{r}$.

If the angular rate named $\Omega_{z}$ is applied along the sensitive axis of resonator, the symmetry axis of hemispherical shell, then the angular rate of resonator is

(13) $$\Omega_{b}={\left[\begin{array}{ccc} 0 & 0 & \Omega_{z} \end{array}\right]}^{T}$$

In Middle Surface Coordinate System, it is

(14) $$\Omega_{m}=C_{b}^{m}\Omega_{b}={\left[\begin{array}{ccc} -\Omega_{z}\sin\alpha & 0 & \Omega_{z}\cos\alpha \end{array}\right]}^{T}$$

In Rotation Coordinate System, the coordinate of point P on middle surface M is

(15) $$r={\left[\begin{array}{ccc} u & v & w+R \end{array}\right]}^{T}$$

According to Coriolis’s Theorem [29] (p. 7), the absolute velocity of point P is

(16) $$\vec{V}=\frac{dr}{dt}+\Omega_{m}\times r=\left[\begin{array}{c} \dot{u}-v\Omega_{z}\cos\alpha \\ \dot{v}+\Omega_{z}\left[(R+w)\sin\alpha +u\cos\alpha \right]\\ \dot{w}-\Omega_{z}u\sin\alpha \end{array}\right]$$

The kinetic energy of hemispherical shell is

(17) $$E_{K}=\frac{1}{2}\int_{R-h}^{R+h}\int_{0}^{2\pi }\int_{0}^{\frac{\pi }{2}}\rho {\vec{V}}^{2}\gamma^{2}\sin\alpha d\alpha d\beta d\gamma$$

where: ρ is the material density of hemispherical shell.

Substituting Equations (6), (8), and (16) into Equation (17), then

(18) $$E_{K}=\frac{1}{2}m_{0}\left({\dot{x}}^{2}+{\dot{y}}^{2}\right)+\frac{1}{2}m_{1}R^{2}\Omega_{z}^{2}+\frac{1}{2}m_{2}\left(\dot{x}y-x\dot{y}\right)+\frac{1}{2}m_{3}\left(x^{2}+y^{2}\right)\Omega_{z}^{2}$$

where: $m_{0}$, $m_{1}$, $m_{2}$, $m_{3}$ can be regarded as equivalent mass, and their computing methods are

(19) $$\{\begin{array}{l} m_{0}=\pi \rho \left(\frac{2}{3}h^{3}+2hR^{2}\right)\int_{0}^{\frac{\pi }{2}}\left(U^{2}+V^{2}+W^{2}\right)\sin\alpha d\alpha \\ m_{1}=2\pi \rho \left(\frac{2}{3}h^{3}+2hR^{2}\right)\int_{0}^{\frac{\pi }{2}}\sin^{3}\alpha d\alpha \\ m_{2}=4\pi \rho \left(\frac{2}{3}h^{3}+2hR^{2}\right)\int_{0}^{\frac{\pi }{2}}\left(U\cos\alpha +W\sin\alpha \right)V\sin\alpha d\alpha \\ m_{3}=\pi \rho \left(\frac{2}{3}h^{3}+2hR^{2}\right)\int_{0}^{\frac{\pi }{2}}\left(V^{2}+U^{2}\cos^{2}\alpha +W^{2}\sin^{2}\alpha +UW\sin2\alpha \right)\sin\alpha d\alpha \end{array}$$

According to Lagrangian Mechanics Principle [30] (pp. 318–323), [31] (pp. 144–145), [16] (p. 16), the Lagrangian Function is

(20) $$L=E_{K}-E_{P}$$

The Lagrangian equations are

(21) $$\{\begin{array}{c} \frac{d}{dt}\left(\frac{\partial L}{\partial \dot{x}}\right)-\frac{\partial L}{\partial x}+\frac{\partial D_{x}}{\partial x}=Q_{x}\\ \frac{d}{dt}\left(\frac{\partial L}{\partial \dot{y}}\right)-\frac{\partial L}{\partial y}+\frac{\partial D_{y}}{\partial y}=Q_{y} \end{array}$$

where: $D_{x}$, $D_{y}$ are the energy dissipation function caused by damping. If the material damping coefficient of axes x, y are $\xi_{x}$, $\xi_{y}$, respectively, then

(22) $$D_{x}=\xi_{x}{\dot{x}}^{2}/2,D_{y}=\xi_{y}{\dot{y}}^{2}/2$$

$Q_{x}$, $Q_{y}$ are the external forces applied on hemispherical shell. If the hemispherical shell is accelerating, then

(23) $$Q_{x}=F_{x}+I_{x},Q_{y}=F_{y}+I_{y}$$

where: $F_{x}$, $F_{y}$ are the electrostatic control forces, and $I_{x}$, $I_{y}$ are inertia forces of hemispherical shell. The expressions of inertia forces are

(24) $$I_{x}=-m_{s}a_{x},I_{y}=-m_{s}a_{y}$$

where: $a_{x}$, $a_{y}$ are the acceleration of hemispherical shell, and $m_{s}$ is the mass of hemispherical shell. The expressions of hemispherical shell mass is

(25) $$m_{s}=\int_{R-h}^{R+h}\int_{0}^{2\pi }\int_{0}^{\frac{\pi }{2}}\rho \gamma^{2}\sin\alpha d\alpha d\beta d\gamma =4\pi \rho h(h^{2}+3r^{2})$$

Combining Equations (10), (11), and (18) to (25), the motion equations of ideal hemispherical shell with perfect dimension and material parameters could be obtained.

(26) $$\{\begin{array}{c} \ddot{x}+2\zeta_{x}\dot{x}+2k_{1}\Omega_{z}\dot{y}+(\omega_{0}^{2}-2k_{2}\Omega_{z}^{2})x+k_{1}{\dot{\Omega }}_{z}y=f_{x}+g_{x}\\ \ddot{y}+2\zeta_{y}\dot{y}-2k_{1}\Omega_{z}\dot{x}+(\omega_{0}^{2}-2k_{2}\Omega_{z}^{2})y-k_{1}{\dot{\Omega }}_{z}x=f_{y}+g_{y} \end{array}$$

where:

(27) $$\{\begin{array}{l} 2\zeta_{x}=\frac{\xi_{x}}{m_{0}},2\zeta_{y}=\frac{\xi_{y}}{m_{0}},\zeta_{x}=\frac{1}{\tau_{x}},\zeta_{y}=\frac{1}{\tau_{y}}\\ f_{x}=\frac{F_{x}}{m_{0}},f_{y}=\frac{F_{y}}{m_{0}},g_{x}=\frac{I_{x}}{m_{0}},g_{y}=\frac{I_{y}}{m_{0}}\\ 2k_{1}=\frac{m_{2}}{m_{0}},2k_{2}=\frac{m_{3}}{m_{0}},\omega_{0}^{2}=\frac{k_{0}}{m_{0}} \end{array}$$

$\zeta_{x}$, $\zeta_{y}$ are material damping ratio. $\tau_{x}$, $\tau_{y}$ are time constants of second-order vibration. $f_{x}$, $f_{y}$ are electrostatic control specific forces. $g_{x}$, $g_{y}$ are inertia specific forces.

When the angular acceleration is omitted ( ${\dot{\Omega }}_{z}=0$ ), the hemispherical shell is vibrating with constant speed ( $f_{x}=f_{y}=g_{x}=g_{y}=0$ ), the damping in axes x, y is the same, and a new complex variable z=x+yi is involved to represent the trajectory of standing wave, then the following Equation (28) can be obtained from Equation (26).

(28) $$\ddot{z}+2(\zeta -k_{1}\Omega_{z}i)\dot{z}+(\omega_{0}^{2}-2k_{2}\Omega_{z}^{2})z=0$$

The solution of Equation (28) is

(29) $$z(t)=e^{-\zeta t}e^{ik_{1}\Omega_{z}t}\left(C_{1}e^{-i\omega_{n}t}+C_{2}e^{i\omega_{n}t}\right)$$

where:

(30) $$\omega_{n}=\sqrt{\omega_{0}^{2}+(k_{1}^{2}-2k_{2})\Omega_{z}^{2}+2k_{1}\zeta \Omega_{z}i-\zeta^{2}}$$

It is clear that damping ratio ζ is responsible for the reduction of standing wave amplitude, and the input angular rate $\Omega_{z}$ is responsible for the precession of standing wave trajectory. The precession factor is

(31) $$K_{g}=k_{1}/2=m_{2}/\left(4m_{0}\right)$$

Partial expression in parenthesis of Equation (29) is just the trajectory of the standing wave, which could also be regarded as a synthesis result of traveling wave in the clockwise direction and traveling wave in counterclockwise direction. If both the damping and external angular rate are omitted, then the second-order free vibration frequency of hemispherical shell is

(32) $$\omega_{0}=\sqrt{k_{0}/m_{0}}$$

**Figure 3:**
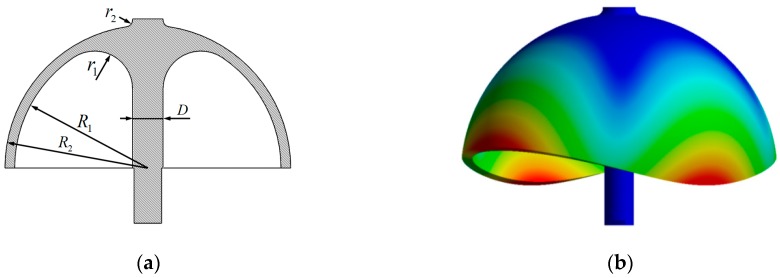
Finite element modal simulation: (**a**) Section view of hemispherical shell resonator; and, (**b**) Finite element modal simulation of hemispherical shell resonator.

The dimension and material parameters of hemispherical shell resonator shown in Figure 3a is listed in Table 1, and the finite element modal simulation is shown in Figure 3b. With the parameters in Table 1, the resonant frequency of the second-order modal calculated by finite element modal simulation is 9195 Hz. The calculation results of Equations (31) and (32) show that the precession factor and the resonant frequency of the second-order modal are −0.2770, 9197 Hz, respectively. The difference of the two second-order resonant frequency that was obtained by Equation (32) and the finite element modal simulation is only 2 Hz.

Reference [21] reported the experimental data of resonant frequency for several real resonators. The actual resonant frequency of resonator numbered 66# is 4853 Hz. According to Equation (32), its theoretical resonant frequency would be 4883 Hz and the relative error is 0.62%, which is within the error range of the Elastic Shell Theory.

Reference [32] (p. 7) reported the performance parameters of HRG158, HRG130, and HRG115 developed by Delco System Operation. The time constants of the three kinds of gyroscopes are 1660 s, 440 s and 110 s respectively. The material damping coefficient of the hemispherical shell resonator is about 1.1 × 10^−5^. According to Equation (27), their theoretical time constants are 1617 s, 433 s, and 108 s, respectively, and the relative errors are 2.59%, 1.59%, and 1.82%, respectively.

As a conclusion, the theoretical results that were obtained by models in this paper are in good agreement with the reported experimental data, which means that the models are reliable and able to reveal the motion features of resonator at a certain level.

### 2.2. Effects of Inherent Frequency Axis and Inherent Damping Axis

Influenced by the mass distribution, the inherent frequencies of hemispherical shell in axes x, y are different. Let the inherent frequencies of axes x, y be $\omega_{x}$, $\omega_{y}$, respectively, then the Equation (26) can be corrected as

(33) $$\{\begin{array}{c} \ddot{x}+2\zeta_{x}\dot{x}+2k_{1}\Omega_{z}\dot{y}+(\omega_{x}^{2}-2k_{2}\Omega_{z}^{2})x+k_{1}{\dot{\Omega }}_{z}y=f_{x}+g_{x}\\ \ddot{y}+2\zeta_{y}\dot{y}-2k_{1}\Omega_{z}\dot{x}+(\omega_{y}^{2}-2k_{2}\Omega_{z}^{2})y-k_{1}{\dot{\Omega }}_{z}x=f_{y}+g_{y} \end{array}$$

**Figure 4:**
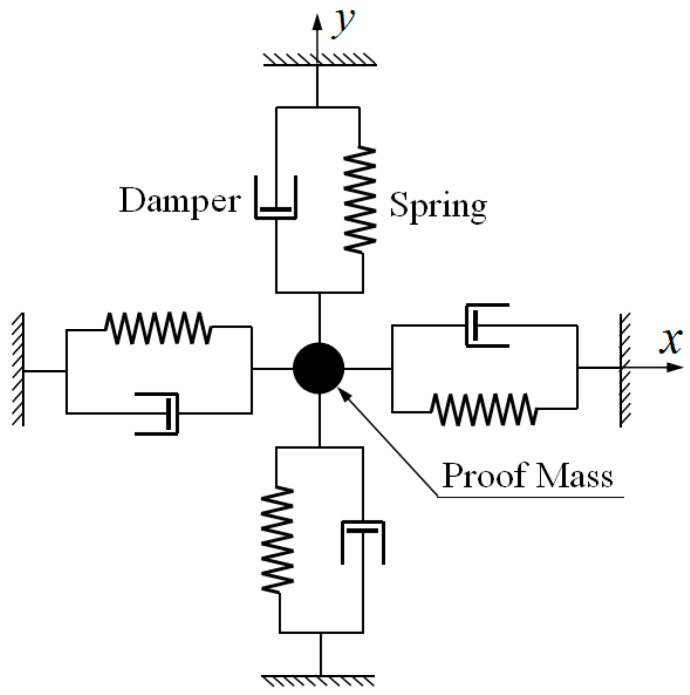
Equivalent mechanical model of ideal hemispherical shell resonator.

Figure 4 shows the equivalent mechanical model of Equation (33). 

**Figure 5:**
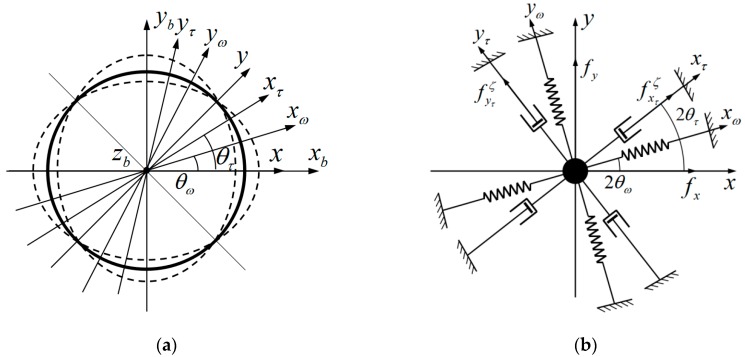
Mechanical model: (**a**) Mechanical model of unideal hemispherical shell resonator; and, (**b**) Equivalent mechanical model of unideal hemispherical shell resonator.

However, in a real HRG system, the inherent frequency axis $x_{\omega }$ will not be coincident with inherent damping axis $x_{\tau }$. Let the axes determined by pickoffs and forcers be x, y. The angles between $x_{\tau }$, x and $x_{\omega }$, x will be $\theta_{\tau }$, $\theta_{\omega }$, respectively [14], as shown in Figure 5a, and Figure 5b shows the equivalent mechanical model.

When the damping forces are omitted, the motion equations of hemispherical shell resonator in axes $x_{\omega }$, $y_{\omega }$ could be written directly by Equation (33):

(34) $$\{\begin{array}{c} {\ddot{x}}_{\omega }+2k_{1}\Omega_{z}{\dot{y}}_{\omega }+(\omega_{x}^{2}-2k_{2}\Omega_{z}^{2})x_{\omega }+k_{1}{\dot{\Omega }}_{z}y_{\omega }=f_{x_{\omega }}+g_{x_{\omega }}\\ {\ddot{y}}_{\omega }-2k_{1}\Omega_{z}{\dot{x}}_{\omega }+(\omega_{y}^{2}-2k_{2}\Omega_{z}^{2})y_{\omega }-k_{1}{\dot{\Omega }}_{z}x_{\omega }=f_{y_{\omega }}+g_{y_{\omega }} \end{array}$$

where:

(35) $$x_{\omega }=x\cos2\theta_{\omega }+y\sin2\theta_{\omega },\;y_{\omega }=-x\sin2\theta_{\omega }+y\cos2\theta_{\omega }\;f_{x_{\omega }}=f_{x}\cos2\theta_{\omega }+f_{y}\sin2\theta_{\omega },\;f_{y_{\omega }}=-f_{x}\sin2\theta_{\omega }+f_{y}\cos2\theta_{\omega }\;g_{x_{\omega }}=g_{x}\cos2\theta_{\omega }+g_{y}\sin2\theta_{\omega },\;g_{y_{\omega }}=-g_{x}\sin2\theta_{\omega }+g_{y}\cos2\theta_{\omega }$$

Substituting Equation (35) into Equation (34), then

(36) $$\{\begin{array}{l} \begin{array}{ll} \ddot{x}+2k_{1}\Omega_{z}\dot{y}+k_{1}{\dot{\Omega }}_{z}y & +\left[\frac{1}{2}\left(\omega_{x}^{2}+\omega_{y}^{2}\right)-2k_{2}\Omega_{z}^{2}\right]x\\ & +\frac{1}{2}\left(\omega_{x}^{2}-\omega_{y}^{2}\right)\left(x\cos4\theta_{\omega }+y\sin4\theta_{\omega }\right)=f_{x}+g_{x} \end{array}\\ \begin{array}{ll} \ddot{y}-2k_{1}\Omega_{z}\dot{x}-k_{1}{\dot{\Omega }}_{z}x & +\left[\frac{1}{2}\left(\omega_{x}^{2}+\omega_{y}^{2}\right)-2k_{2}\Omega_{z}^{2}\right]y\\ & -\frac{1}{2}\left(\omega_{x}^{2}-\omega_{y}^{2}\right)\left(-x\sin4\theta_{\omega }+y\cos4\theta_{\omega }\right)=f_{y}+g_{y} \end{array} \end{array}$$

Next, the effects of damping forces on the motion equations of hemispherical shell resonator is involved. As shown in Figure 5b, the damping specific forces that are caused by equivalent viscous pots on axes $x_{\tau }$, $y_{\tau }$ are $f_{x_{\tau }}^{\zeta }$, $f_{y_{\tau }}^{\zeta }$, respectively, and the time constant are $\tau_{x}$, $\tau_{y}$, respectively. The damping specific forces could be expressed as

(37) $$f_{x_{\tau }}^{\zeta }=-2{\dot{x}}_{\tau }/\tau_{x},f_{y_{\tau }}^{\zeta }=-2{\dot{x}}_{y}/\tau_{y}$$

where:

(38) $$x_{\tau }=x\cos2\theta_{\tau }+y\sin2\theta_{\tau },y_{\tau }=-x\sin2\theta_{\tau }+y\cos2\theta_{\tau }$$

The damping specific forces in axes x, y can be expressed as

(39) $$f_{x}^{\zeta }=f_{x_{\tau }}^{\zeta }\cos2\theta_{\tau }-f_{y_{\tau }}^{\zeta }\sin2\theta_{\tau },f_{y}^{\zeta }=f_{x_{\tau }}^{\zeta }\sin2\theta +f_{y_{\tau }}^{\zeta }\cos2\theta_{\tau }$$

Substituting Equations (37) and (38) into Equation (39), then

(40) $$\{\begin{array}{l} f_{x}^{\zeta }=-\left(\frac{1}{\tau_{x}}+\frac{1}{\tau_{y}}\right)\dot{x}-\left(\frac{1}{\tau_{x}}-\frac{1}{\tau_{y}}\right)\left(\dot{x}\cos4\theta_{\tau }+\dot{y}\sin4\theta_{\tau }\right)\\ f_{y}^{\zeta }=-\left(\frac{1}{\tau_{x}}+\frac{1}{\tau_{y}}\right)\dot{y}+\left(\frac{1}{\tau_{x}}-\frac{1}{\tau_{y}}\right)\left(-\dot{x}\sin4\theta_{\tau }+\dot{y}\cos4\theta_{\tau }\right) \end{array}$$

Equation (36) can be corrected further, as

(41) $$\{\begin{array}{l} \begin{array}{l} \ddot{x}+2k_{1}\Omega_{z}\dot{y}+k_{1}{\dot{\Omega }}_{z}y+\left(\frac{1}{\tau_{x}}+\frac{1}{\tau_{y}}\right)\dot{x}+\left(\frac{1}{\tau_{x}}-\frac{1}{\tau_{y}}\right)\left(\dot{x}\cos4\theta_{\tau }+\dot{y}\sin4\theta_{\tau }\right)\\ \;+\left[\frac{1}{2}\left(\omega_{x}^{2}+\omega_{y}^{2}\right)-2k_{2}\Omega_{z}^{2}\right]x+\frac{1}{2}\left(\omega_{x}^{2}-\omega_{y}^{2}\right)\left(x\cos4\theta_{\omega }+y\sin4\theta_{\omega }\right)=f_{x}+g_{x} \end{array}\\ \begin{array}{l} \ddot{y}-2k_{1}\Omega_{z}\dot{x}-k_{1}{\dot{\Omega }}_{z}x+\left(\frac{1}{\tau_{x}}+\frac{1}{\tau_{y}}\right)\dot{y}-\left(\frac{1}{\tau_{x}}-\frac{1}{\tau_{y}}\right)\left(-\dot{x}\sin4\theta_{\tau }+\dot{y}\cos4\theta_{\tau }\right)\\ \;+\left[\frac{1}{2}\left(\omega_{x}^{2}+\omega_{y}^{2}\right)-2k_{2}\Omega_{z}^{2}\right]y-\frac{1}{2}\left(\omega_{x}^{2}-\omega_{y}^{2}\right)\left(-x\sin4\theta_{\omega }+y\cos4\theta_{\omega }\right)=f_{y}+g_{y} \end{array} \end{array}$$

The motion equations shown in Equation (41) contain the angle parameters between the inherent feature axes and forcer-pickoff axes, and they can be used within the analysis of free vibration and forced vibration of resonator affected by inherent feature axes. The expression form of motion equations shown in Equation (41) is similar to motion equations of Generic Vibratory Gyroscope that are given by reference [14]. Nevertheless, when all of the parameters of the hemispherical shell resonator are determined, the coefficients in Equation (41) can be calculated one by one, according to Equation (27). In contrast, the coefficients of equations given by reference [14] can be only determined by experiences, even endless attempts. Obviously, Equation (41) is more efficient and reliable in establishing the connection between the real HRG systems and mathematical models, and it is more suitable for quantitative analysis than equations that are given by reference [14]. 

### 2.3. Effects of External Control Force

Assuming that the amount of control capacitors providing electrostatic forces around the resonator is N, and the $\theta_{c}(i)$ is the angle of i*-th* capacitor around the symmetry axis of the hemispherical shell. After the applied voltage, the i*-th* capacitor will generate electrostatic attraction force $F_{i}^{e}$ along the radial direction of hemispherical shell. The components of $F_{i}^{e}$ on axis x and axis y are

(42) $$F_{i}^{x}=F_{i}^{e}\cos2\theta_{c}(i),F_{i}^{y}=F_{i}^{e}\sin2\theta_{c}(i)$$

Subsequently, the total electrostatic control forces can be expressed as

(43) $$F_{x}=\sum_{i=1}^{N}F_{i}^{x},F_{y}=\sum_{i=1}^{N}F_{i}^{y}$$

According to Equation (27), the following Equation (44) can be deduced

(44) $$\{\begin{array}{l} f_{x}=\frac{1}{m_{0}}F_{x}=\frac{1}{m_{0}}\sum_{i=1}^{N}F_{i}^{e}\cos2\theta_{c}(i)=\sum_{i=1}^{N}f_{i}^{e}\cos2\theta_{c}(i)\\ f_{y}=\frac{1}{m_{0}}F_{y}=\frac{1}{m_{0}}\sum_{i=1}^{N}F_{i}^{e}\sin2\theta_{c}(i)=\sum_{i=1}^{N}f_{i}^{e}\sin2\theta_{c}(i) \end{array}$$

where:

(45) $$f_{i}^{e}=F_{i}^{e}/m_{0}$$

Substituting Equations (44) into Equation (41), the final Equation (46) would be obtained.

(46) $$\{\begin{array}{c} \begin{array}{cc} \ddot{x} & +\left[2k_{1}\Omega_{z}+\left(\frac{1}{\tau_{x}}-\frac{1}{\tau_{y}}\right)\sin4\theta_{\tau }\right]\dot{y}+\left[\left(\frac{1}{\tau_{x}}+\frac{1}{\tau_{y}}\right)+\left(\frac{1}{\tau_{x}}-\frac{1}{\tau_{y}}\right)\cos4\theta_{\tau }\right]\dot{x}\\ & +\left[k_{1}{\dot{\Omega }}_{z}+\frac{1}{2}\left(\omega_{x}^{2}-\omega_{y}^{2}\right)\sin4\theta_{\omega }\right]y+\left[\frac{1}{2}\left(\omega_{x}^{2}+\omega_{y}^{2}\right)-2k_{2}\Omega_{z}^{2}+\frac{1}{2}\left(\omega_{x}^{2}-\omega_{y}^{2}\right)\cos4\theta_{\omega }\right]x\\ & =\sum_{i=1}^{N}f_{i}^{e}\cos2\theta_{c}(i)+g_{x} \end{array}\\ \begin{array}{cc} \ddot{y} & -\left[2k_{1}\Omega_{z}-\left(\frac{1}{\tau_{x}}-\frac{1}{\tau_{y}}\right)\sin4\theta_{\tau }\right]\dot{x}+\left[\left(\frac{1}{\tau_{x}}+\frac{1}{\tau_{y}}\right)-\left(\frac{1}{\tau_{x}}-\frac{1}{\tau_{y}}\right)\cos4\theta_{\tau }\right]\dot{y}\\ & -\left[k_{1}{\dot{\Omega }}_{z}-\frac{1}{2}\left(\omega_{x}^{2}-\omega_{y}^{2}\right)\sin4\theta_{\omega }\right]x+\left[\frac{1}{2}\left(\omega_{x}^{2}+\omega_{y}^{2}\right)-2k_{2}\Omega_{z}^{2}-\frac{1}{2}\left(\omega_{x}^{2}-\omega_{y}^{2}\right)\cos4\theta_{\omega }\right]y\\ & =\sum_{i=1}^{N}f_{i}^{e}\sin2\theta_{c}(i)+g_{y} \end{array} \end{array}$$

Distributed capacitor electrostatic control forces are introduced in Equation (46), which is suitable for vibration characteristics analysis of the hemispherical shell resonator with multi-capacitor control, such as HRG130Y containing 32 uniform distribution control electrodes [1]. When compared with Equation (41), Equation (46) is closer to the real HRG system. 

## 3. Excitation and Detection Models

In this section, the mathematical models of the exciting system and detecting system of flat-electrode HRG are established. Firstly, the unique structures of the flat-electrode HRG capacitor are introduced, and the assemble errors and parameters that will affect the capacitance of excitation and detection capacitors are pointed out. Secondly, the models of excitation and detection capacitors involving assemble errors and parameters are deduced. Next, the Lagrangian Mechanics Principle establishes the exciting force models of the hemispherical shell resonator from voltage on capacitors to electrostatic forces. Finally, the detection models of resonator from displacements of the resonator lip to voltage on equivalent resistor are deduced. The modeling methods that are mentioned in this section are also suitable for all kinds of HRG with different capacitor structures.

### 3.1. Capacitor Structure

**Figure 6:**
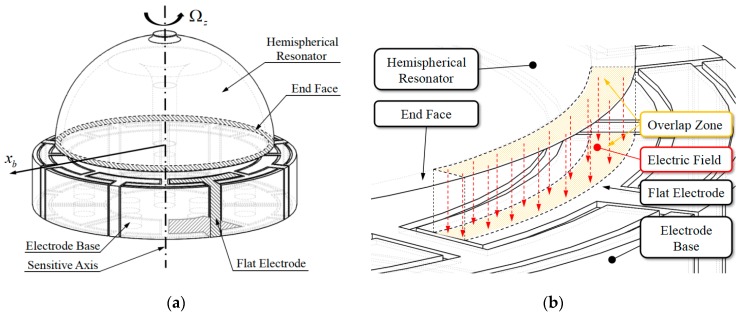
Flat-electrode Hemispherical Resonator Gyro (HRG): (**a**) Dominant structure of flat-electrode HRG; and, (**b**) Excitation and detection capacitor.

Figure 6a shows the dominant structure of flat-electrode HRG. The hemispherical shell resonator is installed at the centre of disc-shaped electrode base. The inner surface and end face of resonator are metalized with thin platinum coating. The surfaces of electrode base are also metalized with a thin platinum coating engraved with several non-conductive grooves and forming eight equidistribution flat electrodes. The overlap zone of resonator end face and flat electrode composes the excitation and detection capacitor, as shown in Figure 6b. When changing voltage is applied to the capacitor, a corresponding electric field is also established.

Ideally, the end face of resonator is parallel with the flat electrode, and the width of the flat electrode far outweighs the thickness of resonator. According to reference [8], the vibration amplitude of resonator lip along the direction of resonator sensitive axis is in a range of from 0.5 μm to 1 μm, and the vibration amplitude of resonator lip along the radial direction of resonator lip will be in a range of from 1 μm to 2 μm, which is far less than the difference between the width of the flat electrode and the thickness of resonator. It means that the projection of resonator end face on electrode base will never exceed the flat electrode zone when the resonator is vibrating, the electrode area of excitation and detection capacitors will be almost constant, and the capacitance variation will be directly caused by the gap variation between the end face of resonator and electrode base. In a nutshell, the capacitance variation is caused by the gap variation, and the electrode area is almost constant.

The structural feature of the flat-electrode HRG determines that its performance will be almost unaffected by concentric error between resonator and electrode base caused by the assembly procedure. On the contrary, the concentric error will severely affect the performance of classical three-dimensional electrode HRG. As a result, this is another great advantage of the flat-electrode HRG.

**Figure 7:**
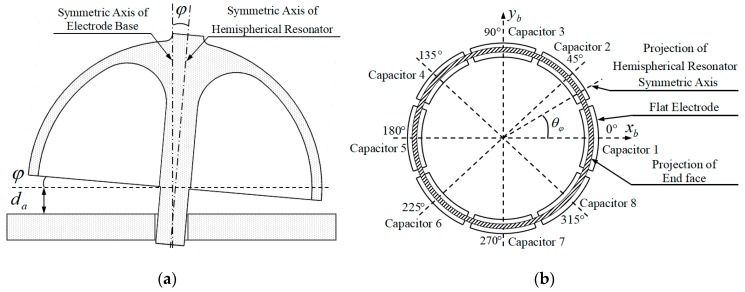
Assemble errors and parameters caused by assemble procedure: (**a**) Inclination angle of hemispherical resonator symmetric axis and average gap between end face of resonator lip and electrode base; and, (**b**) Direction angle of resonator symmetric axis.

Actually, the assembly procedure of resonator will not be perfect, and corresponding assembly errors and parameters will surely exist. As shown in Figure 7a, the inclination angle between resonator symmetric axis and electrode base symmetric axis is ϕ, one of the assembly errors. The average gap between the end face of resonator lip and electrode base is $d_{a}$, one assembly parameter. As shown in Figure 7b, the angle between projection of resonator symmetric axis and axis $x_{b}$ is direction angle $\theta_{\phi }$, one of the assembly errors. ϕ, $d_{a}$, and $\theta_{\phi }$ are responsible for the first-harmonic distribution of gap between the lip and the base, and for the difference between the excitation and detection capacitors. Unfortunately, it is nearly impossible to directly measure these errors or parameters. The only way to obtain the information relies on identification method based on static capacitance of the excitation and detection capacitor, which will be detailed in the next section.

### 3.2. Capacitor Model

**Figure 8:**
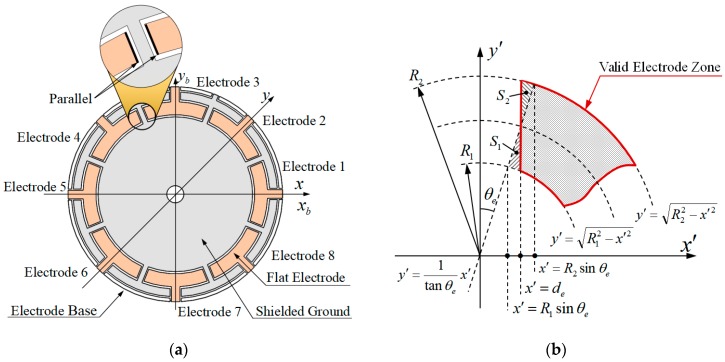
Electrode of flat-electrode HRG: (**a**) Top view of electrode base; and, (**b**) Enlarged view of part Valid Electrode Zone.

As shown in Figure 8a, to ensure the approximate equidistribution of charge on electrodes, the border between the two electrodes is parallel to a distance $2d_{e}$. However, this design makes it hard to establish the capacitor model. To get around this, the conception of Equivalent Interval Angle is introduced. As shown in Figure 8b, part of the flat electrode is enlarged. The zone that is surrounded by solid line is the overlap zone of resonator end face and flat electrode, named the Valid Electrode Zone. Two adjacent electrodes are separated by non-conductive grooves with a parallel border, and the width is $2d_{e}$. To simplify the modeling process, the two adjacent electrodes are considered to be separated by non-conductive grooves with sector border, and the central angle is $2\theta_{e}$, named the Equivalent Interval Angle. If the shadow areas $S_{1}$, $S_{2}$ in Figure 8b are the same, then the area of the Valid Electrode Zone that is separated by the parallel border is the same with the area of Valid Electrode Zone separated by imaginary sector border. It is clear that the effect of the capacitor model that was established with the sector border is nearly the same with the one with parallel border.

Half of the Equivalent Interval Angle can be calculated by following Equation (47)

(47) $$\{\begin{array}{c} S_{1}=\int_{R_{1}\sin\theta_{e}}^{d_{e}}\left(\frac{1}{\tan\theta_{e}}x^{\prime }-\sqrt{R_{1}^{2}-{x^{\prime }}^{2}}\right)dx^{\prime }\\ S_{2}=\int_{d_{e}}^{R_{2}\sin\theta_{e}}\left(\sqrt{R_{2}^{2}-{x^{\prime }}^{2}}-\frac{1}{\tan\theta_{e}}x^{\prime }\right)dx^{\prime } \end{array},\;S_{1}-S_{2}=0$$

The Border Angle of each Valid Electrode Zone for eight-electrode HRG in axes $x_{b}y_{b}$ would be

(48) $$\{\begin{array}{c} \theta_{r}(i)=\theta_{c}(i)+\pi /8-\theta_{e}\\ \theta_{l}(i)=\theta_{c}(i)-\pi /8+\theta_{e} \end{array}$$

where: $\theta_{r}(i)$, $\theta_{l}(i)$ are the Right Border Angle and Left Border Angle of the *i*-th electrode, respectively. $\theta_{c}(i)$ is the Centre Angle of the *i*-th electrode.

In summary, according to all of the details mentioned above, the capacitance of a excitation and detection capacitor can be expressed as

(49) $$C_{F}=\int_{\theta_{L}}^{\theta_{R}}\frac{1}{4\pi k}\frac{\left(R_{2}^{2}-R_{1}^{2}\right)/2}{d_{F}}d\theta$$

where: $\theta_{R}$, $\theta_{L}$ are the Right Border Angle and Left Border Angle of excitation and detection capacitor. *d_F_* is the gap of capacitor. *k* is Coulomb’s constant. 

When the hemispherical shell resonator is stationary, the gap between end face of resonator lip and electrode base could be expressed as

(50) $$d_{F}=d_{a}-\phi R\cos(\theta -\theta_{\phi })$$

Substituting *α = π/2*, *β = θ* into Equation (6), when resonator is vibrating the gap, would be

(51) $$d_{F}=d_{a}-\phi R\cos(\theta -\theta_{\phi })-\left(x\cos2\theta +y\sin2\theta \right)$$

Let $\theta_{R}=\theta_{r}(i)$, $\theta_{l}=\theta_{l}(i)$, according to Equations (49), (50), and (51), the capacitance of the capacitor could be calculated when the resonator is vibrating or stationary. 

In some circumstances, it is not necessary to get such high-precision capacitance calculation of every excitation and detection capacitor, and Equation (49) can be simplified by the following methods. Here, the gap variation caused by θ, within a capacitor, is omitted. It means that, for a certain excitation and detection capacitor, $d_{F}$ is independent, with θ. Let $\Delta \theta_{F}(i)=\theta_{r}(i)-\theta_{l}(i)$ represent the span of each capacitor. In consideration of the equidistribution of capacitors, $\Delta \theta_{F}$ is a constant and independent with θ. Subsequently, Equation (49) can be rewritten as

(52) $$C_{F}=K_{F}/d_{F}$$

where:

(53) $$K_{F}=\frac{\Delta \theta_{F}\left(R_{2}^{2}-R_{1}^{2}\right)}{8\pi k}$$

When the hemispherical shell resonator is stationary, the gap of *i*-th capacitor will be

(54) $$d_{F}(i)=d_{a}-\phi R\cos\left[\theta_{c}(i)-\theta_{\phi }\right]$$

When the resonator is vibrating, the gap of *i*-th capacitor will be

(55) $$d_{F}(i)=d_{a}-\phi R\cos\left[\theta_{c}(i)-\theta_{\phi }\right]-\left[x\cos2\theta_{c}(i)+y\sin2\theta_{c}(i)\right]$$

The capacitance of *i*-th capacitor will be

(56) $$\begin{array}{ll} C_{F}(i) & =\frac{K_{F}}{d_{a}-\phi R\cos\left[\theta_{c}(i)-\theta_{\phi }\right]-\left[x\cos2\theta_{c}(i)+y\sin2\theta_{c}(i)\right]}\\ & \approx K_{F}\left\{\frac{1}{d_{a}-\phi R\cos\left[\theta_{c}(i)-\theta_{\phi }\right]}-\frac{1}{{\left[d_{a}-\phi R\cos\left[\theta_{c}(i)-\theta_{\phi }\right]\right]}^{2}}\left[x\cos2\theta_{c}(i)+y\sin2\theta_{c}(i)\right]\right\} \end{array}$$

In Equation (56), the capacitance of capacitor i is simplified as linear function of x, y. In an eight-electrode HRG, for a certain capacitor, only one of x, y in Equation (56) will be preserved. For instance, the capacitance of capacitor 3 and 4 are

(57) $$\{\begin{array}{l} C_{F}(3)=K_{F}\left\{{\left(d_{a}-\phi R\sin\theta_{\phi }\right)}^{-1}+{\left(d_{a}-\phi R\sin\theta_{\phi }\right)}^{-2}x\right\}\\ C_{F}(4)=K_{F}\left\{{\left(d_{a}-\phi R\sin\left(\theta_{\phi }-\pi /4\right)\right)}^{-1}+{\left[d_{a}-\phi R\sin\left(\theta_{\phi }-\pi /4\right)\right]}^{-2}y\right\} \end{array}$$

### 3.3. Excitation Model

According to Lagrangian Mechanics Principle [30] (pp. 318–323), [31] (pp. 144–145), [16] (p. 16), the electrostatic force [33] (p. 68) between the resonator lip and flat electrode within a certain excitation and detection capacitor is

(58) $$F_{c}=-\frac{\partial E_{c}}{\partial d_{F}}$$

where: $E_{c}$ is the electric potential energy stored by capacitor, and

(59) $$E_{c}=\frac{1}{2}C_{F}U_{F}^{2}$$

where: $U_{F}$ is voltage applied to the capacitor.

As to ith capacitor, when the resonator is vibrating, the electrostatic force would be

(60) $$\begin{array}{ll} F_{c}(i) & =-\frac{U_{F}^{2}}{2}\frac{\partial C_{F}(i)}{\partial d_{F}}=-\frac{U_{F}^{2}}{2}\cdot \frac{\left(R_{2}^{2}-R_{1}^{2}\right)}{8\pi k}\int_{\theta_{l}(i)}^{\theta_{r}(i)}\frac{1}{d_{F}^{2}}d\theta \\ & =-\frac{U_{F}^{2}}{2}\cdot \frac{\left(R_{2}^{2}-R_{1}^{2}\right)}{8\pi k}\int_{\theta_{l}(i)}^{\theta_{r}(i)}{\left\{d_{a}-\phi R\cos\left[\theta -\theta_{\phi }\right]-\left[x\cos2\theta +y\sin2\theta \right]\right\}}^{-2}d\theta \end{array}$$

If the simplification methods that were used in Equations (52) to (56) are also involved here, then the expression of electrostatic force could be simplified as

(61) $$\begin{array}{ll} F_{c}(i) & \approx -\frac{U_{F}^{2}}{2}\cdot \frac{\left(R_{2}^{2}-R_{1}^{2}\right)}{8\pi k}\Delta \theta_{F}{\left\{d_{a}-\phi R\cos\left[\theta_{c}(i)-\theta_{\phi }\right]-\left[x\cos2\theta_{c}(i)+y\sin2\theta_{c}(i)\right]\right\}}^{-2}\\ & =-\frac{1}{2}K_{F}U_{F}^{2}{\left\{d_{a}-\phi R\cos\left[\theta_{c}(i)-\theta_{\phi }\right]-\left[x\cos2\theta_{c}(i)+y\sin2\theta_{c}(i)\right]\right\}}^{-2}\\ & =-\frac{1}{2}K_{F}U_{F}^{2}\left\{\begin{array}{l} \frac{1}{{\left[d_{a}-\phi R\cos\left[\theta_{c}(i)-\theta_{\phi }\right]\right]}^{2}}\\ +\frac{2}{{\left[d_{a}-\phi R\cos\left[\theta_{c}(i)-\theta_{\phi }\right]\right]}^{3}}\left[x\cos2\theta_{c}(i)+y\sin2\theta_{c}(i)\right] \end{array}\right\} \end{array}$$

In Equation (61), the electrostatic force is simplified as linear function of x, y. In eight-electrode HRG, for a certain capacitor, only one of x, y in Equation (61) will be preserved. For instance, the electrostatic force of capacitor 1 and 2 are

(62) $$\{\begin{array}{l} F_{c}(1)=-\frac{1}{2}K_{F}U_{F}^{2}\left\{{\left(d_{a}-\phi R\cos\theta_{\phi }\right)}^{-2}+2{\left(d_{a}-\phi R\cos\theta_{\phi }\right)}^{-3}x\right\}\\ F_{c}(2)=-\frac{1}{2}K_{F}U_{F}^{2}\left\{{\left[d_{a}-\phi R\cos\left(\pi /4-\theta_{\phi }\right)\right]}^{-2}+2{\left[d_{a}-\phi R\cos\left(\pi /4-\theta_{\phi }\right)\right]}^{-3}y\right\} \end{array}$$

The relationship between electrostatic force $F_{c}(i)$ and external control force $F_{i}^{e}$ is $F_{i}^{e}=-F_{c}(i)$.

### 3.4. Detection Model

**Figure 9:**
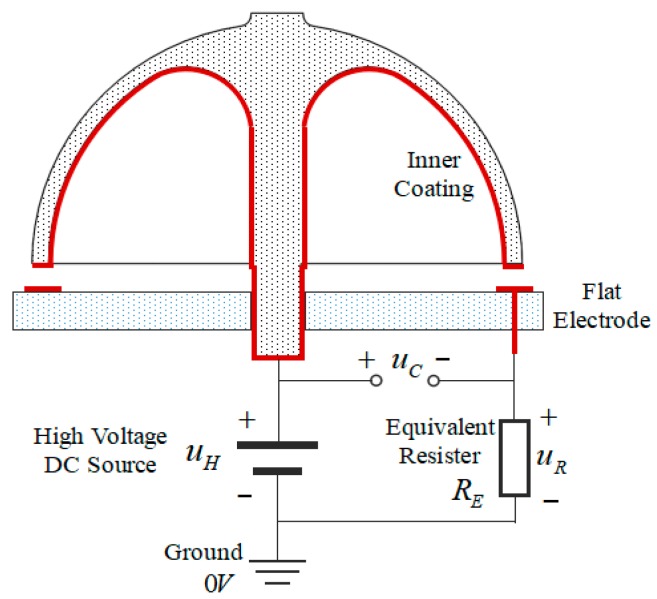
Schematic of signal converter.

When compared with the Excitation Model, the Detection Model of hemispherical shell resonator seems to be more complex. It is because the direct response of HRG with harmonic control forces is the capacitance variation of the detection capacitor. This state could not be directly measured, and it has to be converted to the voltage variation on detection flat electrodes. Hence, the following schematic that is shown in Figure 9 is adopted to convert the capacitance variation of detection capacitor to the voltage variation on detection flat electrodes [34].

The inner platinum coating of the resonator is connected with positive anode of a DC source with high voltage $u_{H}$, the cathode of DC source and one pin of equivalent resistor are both connected with the ground, and the other pin of the equivalent resistor is connected with a certain flat electrode. In result, the voltage from the anode of the DC source to the flat electrode will be the voltage of excitation and detection capacitor $u_{C}$, and it can be determined by voltage on the equivalent resistor $u_{R}$. It means that the vibration response of HRG is no longer the capacitance variation of detection capacitor, but the voltage on equivalent resistor.

The expression of $u_{R}$ in Figure 9 is

(63) $${\dot{u}}_{R}=\frac{u_{H}}{C_{F}}{\dot{C}}_{F}-u_{R}\left(\frac{1}{R_{E}}+\frac{1}{C_{F}}{\dot{C}}_{F}\right)$$

If a certain capacitor is selected, the expression of $C_{F}$ could be Equation (49) or (56), depending on the required precision. The practical equivalent resistor usually consisted of operational amplifier circuits [28] (p. 46).

## 4. Identification of Assemble Errors and Parameters

As mentioned in last section, the assembly procedure of hemispherical shell resonator with electrode base will not be perfect, and corresponding assemble errors and parameters, such as inclination angle ϕ, average gap $d_{a}$, and direction angle $\theta_{\phi }$, will surely exist. In this section, an identification method of assemble errors and parameters is proposed based on the principle of minimum square sum of errors, and a three-phase identification scheme is designed. Under the guidance of the three-phase identification scheme, the average gap $d_{a}$ should be identified before estimating inclination angle ϕ and direction angle $\theta_{\phi }$ of inclination. This is the last piece of the puzzle, and according to this method, arguments in the equations that are mentioned in the previous section would finally be determined, which means that all of the required information in excitation and detection models are provided. Besides, not only the identification and analysis for assembly errors and parameters will facilitate the comprehension of HRG error mechanism and compensation for the improvement of HRG performance, but also the evaluation and improvement of HRG manufacturing technology.

### 4.1. Approximate Identification Model

Based on Equations (49) and (50), the static capacitor model could be expressed as

(64) $$C_{F}=\int_{\theta_{L}}^{\theta_{R}}\frac{1}{4\pi k}\frac{\left(R_{2}^{2}-R_{1}^{2}\right)/2}{d_{a}-\phi R\cos(\theta -\theta_{\phi })}d\theta =\frac{\left(R_{2}^{2}-R_{1}^{2}\right)}{8\pi k}\int_{\theta_{L}}^{\theta_{R}}\frac{1}{d_{a}-\phi R\cos(\theta -\theta_{\phi })}d\theta$$

Unfortunately, there is a trigonometric function in the integrand of Equation (64), which complicates the final expression of $C_{F}$, and it makes it hard to be used in parameter identifications. The approximate static capacitance analytic expression can be given as Equation (65), which is more suitable for the identification of assemble errors and parameters than Equation (64).

(65) $${\tilde{C}}_{F}(i)=\lambda_{1}\left\{{\left[1+\lambda_{2}\cos[\theta_{c}(i)-\theta_{\phi }]\right]}^{\lambda_{3}\phi }-1\right\}+\lambda_{4}$$

where: $\lambda_{1}$, $\lambda_{2}$, $\lambda_{3}$, and $\lambda_{4}$ are the approximate static capacitor model parameters that are needed to be determined later, and $\lambda_{4}$ is exactly the average capacitance of excitation and detection capacitors. 

**Figure 10:**
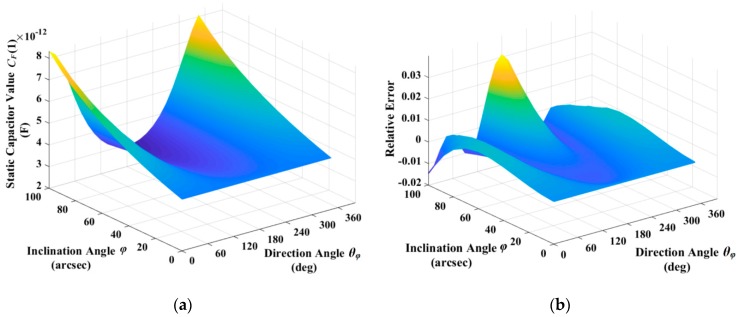

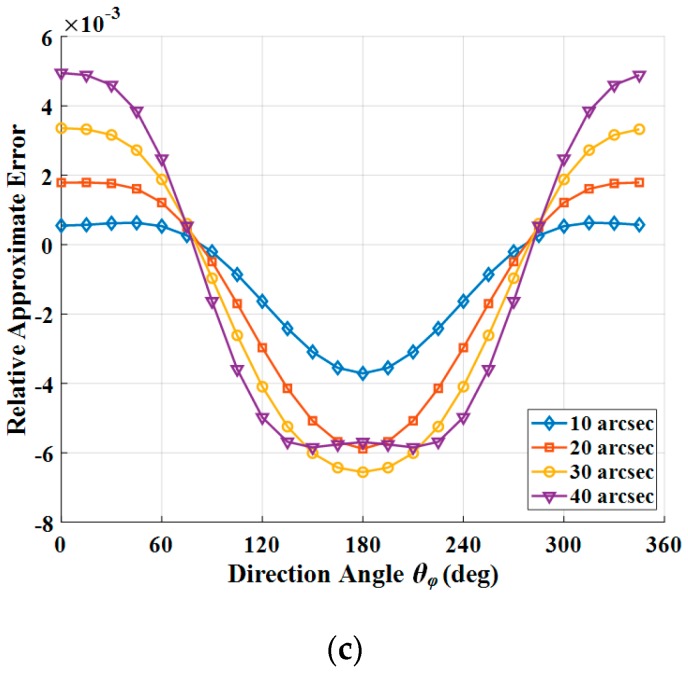
Static capacitor simulation: (**a**) Ideal static capacitance of capacitor 1; (**b**) Relative error of capacitor 1; and, (**c**) Relative error of capacitor 1 when inclination angle is 0″, 10″, 20″, and 30″.

When the parameters that are listed in Table 2 are adopted, the ideal static capacitance of capacitor 1 is shown in Figure 10a, and the relative error δE defined as Equation (66) is shown in Figure 10b. For the typical inclination angle, 0″, 10″, 20″, and 30″, the relative error δE is shown in Figure 10c. The maximal relative error between Equations (64) and (65) is about 3% within the simulation situations that are listed in Table 2.

(66) $$\delta E=\left({\tilde{C}}_{F}-C_{F}\right)/C_{F}$$

### 4.2. Identification Method of Assemble Errors and Parameters

As shown in Equation (64), the capacitance of parallel-plate capacitor is an inverse function of the capacitor gap, and the gap has the greatest influence on capacitance. In view of this consideration, a three-phase identification scheme is designed. Firstly, the average gap is identified by the average static capacitance of all excitation and detection capacitors. Secondly, the parameters of Equation (65) are identified to obtain the approximate static model of capacitors. Finally, the assemble errors and parameters are identified by the approximate static model of capacitor.

According to Equation (65), the sum of capacitances of all capacitors for eight-electrode HRG with small $\lambda_{3}\cdot \phi$ and $\lambda_{2}$ is

(67) $$\sum_{i=1}^{8}{\tilde{C}}_{F}(i)=\lambda_{1}\sum_{i=1}^{8}\left\{{\left[1+\lambda_{2}\cos[\theta_{c}(i)-\theta_{\phi }]\right]}^{\lambda_{3}\phi }-1\right\}+8\lambda_{4}\approx 8\lambda_{4}$$

It means that $\lambda_{4}$ is exactly the average of all capacitance. Let $C_{M}(i)$ be the practical measurement of the *i*-th capacitor and the estimated value of average gap is

(68) $${\hat{d}}_{a}=\Delta \theta_{F}\left(R_{2}^{2}-R_{1}^{2}\right)/\left[\pi k\sum_{i=1}^{8}C_{M}(i)\right]$$

Let ϕ(m), $\theta_{\phi }(l)$ be the value of inclination angle and direction angle, respectively, and let $C_{Z}(m,l)$ be the ideal simulation capacitance related ϕ(m), $\theta_{\phi }(l)$. According to Equation (64), the expression of $C_{Z}(m,l)$ is

(69) $$C_{Z}(m,l)=\frac{\left(R_{2}^{2}-R_{1}^{2}\right)}{8\pi k}\int_{\theta_{L}}^{\theta_{R}}\frac{1}{d_{a}-\phi (m)R\cos\left[\theta -\theta_{\phi }(l)\right]}d\theta$$

In order to identify $\lambda_{1}$, $\lambda_{2}$, $\lambda_{3}$, $\lambda_{4}$, an objective function $J_{\lambda }$ could be established as Equation (70) based on the principle of the minimum square sum of errors.

(70) $$J_{\lambda }=\sum_{m=1}^{M}\sum_{l=1}^{L}{\left\{C_{Z}(m,l)-\lambda_{1}\left\{{\left[1+\lambda_{2}\cos[\theta_{c}(i)-\theta_{\phi }(l)]\right]}^{\lambda_{3}\phi (m)}-1\right\}-\lambda_{4}\right\}}^{2}$$

where: M, L are the total amount of inclination angle and direction angle used in simulation, respectively.

When the minimum of $J_{\lambda }$ is obtained, parameters $\lambda_{1}$, $\lambda_{2}$, $\lambda_{3}$, $\lambda_{4}$ in Equation (65) are immediately obtained, and the relative error between Equation (65) and Equation (64) will be minimized. The minimum problem of $J_{\lambda }$ could be solved by iteration based on the Nelder–Mead Simplex Method [35], and the initial value of iteration could be $\lambda_{1}$ = 2 × 10^−12^F, $\lambda_{2}$ = 0.09, $\lambda_{3}$ = 2 × 10^4^, and $\lambda_{4}$ = 5 × 10^−12^F. When the approximate static model of capacitors is obtained by Equation (70), the following objective function $J_{\phi }$ could be also established by the same principle.

(71) $$J_{\phi }=\sum_{i=1}^{8}{\left\{C_{M}(i)-\lambda_{1}\left\{{\left[1+\lambda_{2}\cos[\theta_{c}(i)-{\hat{\theta }}_{\phi }]\right]}^{\lambda_{3}\hat{\phi }}-1\right\}-\lambda_{4}\right\}}^{2}$$

When the minimum of $J_{\phi }$ is obtained, $\hat{\phi }$ and ${\hat{\theta }}_{\phi }$ will be the estimation of inclination angle and direction angle, respectively.

In summary, the identification of assemble errors and parameters could be accomplished by Equations (68) to (71) in three steps.

### 4.3. Analysis of Simulation Results

Here, the availability and accuracy of the parameter identification method mentioned above is analyzed by simulation, and the assumed conditions are listed below:

After the assemble procedure, a gap nearly 10 μm to 20 μm, as shown in Figure 6a between resonator lip and electrode base, will exist [8]. Therefore, the typical average gap assumed is 15 μm. Assuming that the change ratio of gap between resonator and electrode base at some points is 0.05 to 0.10, then the range of typical inclination angle will not exceed the range of 10″ to 40″. The measuring standard deviation of static capacitance is assumed to be 0.005pF, and the mean of measurement is zero.

**Figure 11:**
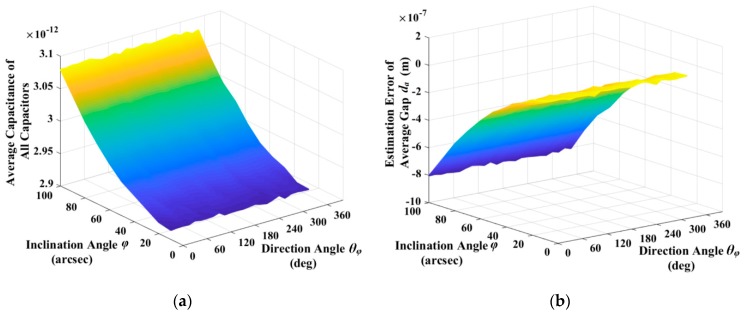
Average capacitance of all capacitors and estimation error of average gap $d_{a}$: (**a**) Average capacitance of all capacitors with different inclination angle and direction angle; and, (**b**) Estimation error of average gap with different inclination angle and direction angle.

Simulations, according Equation (64) and the assumptions mentioned above can obtain the static capacitance of all eight excitation and detection capacitors of flat-electrode HRG. Figure 11a shows the average capacitance of eight capacitors with different inclination angle, direction angle, and typical average gap. As shown in Figure 11a, the average capacitance is independent with the direction angle, and it increases slowly with inclination angle. The change, within the range of typical inclination angle, is just 2.3021 × 10^−2^ pF, which is less than 1% of the static capacitance in this range, within the approximate error range of Equation (65). As a result, it is reasonable to estimate the average gap firstly, according to Equation (68), and the estimation error of average gap $d_{a}$ is shown in Figure 11b. As shown in Figure 11b, the estimation error of average gap is independent with direction angle, and it increases slowly with inclination angle. 

**Figure 12:**
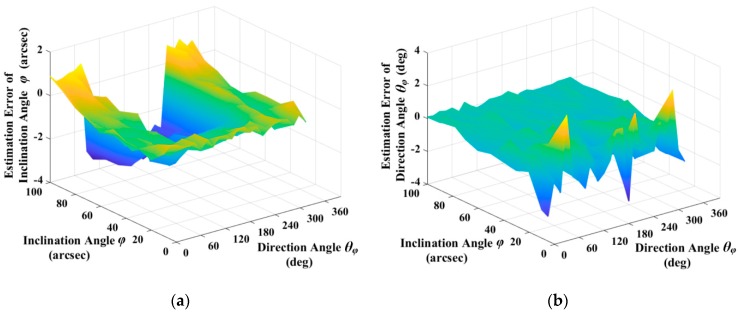
Estimation error of Inclination Angle ϕ and Direction Angle $\theta_{\phi }$: (**a**) Estimation error of inclination angle with different inclination angle and direction angle; and, (**b**) Estimation error of direction angle with different inclination angle and direction angle.

After the identification of average gap, the parameters in the approximate static model of excitation and detection capacitors could be obtained by Equation (70), and the estimation result of inclination angle ϕ and direction angle $\theta_{\phi }$ could be subsequently obtained by Equation (71). Figure 12a,b show the estimation results. As shown in Figure 12a, the estimation error of inclination angle increases slowly with inclination angle, and its sign is related with direction angle due to the periodicity of trigonometric functions. In the range of 90° to 270°, the sign of the estimation error is negative, beyond that is positive. However, the absolute value of estimation error within each range is independent with direction angle. As shown in Figure 12b, the estimation error of direction angle is independent with direction angle and it decreases with inclination angle, and the direction angle at the maximum estimation error of direction angle is determined by the random noise that is involved in the simulation.

**Figure 13:**
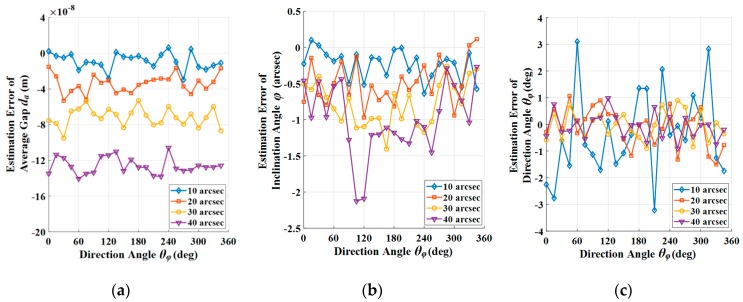
Estimation error of assemble errors and parameters: (**a**) Estimation error of average gap with typical inclination angle and different direction angle; (**b**) Estimation error of inclination angle with typical inclination angle and different direction angle; and, (**c**) Estimation error of direction angle with typical inclination angle and different direction angle.

The estimation errors of average gap, inclination angle, and direction angle are shown in Figure 13a, Figure 13b, and Figure 13c respectively. With a typical inclination angle 20″, the estimation error of average gap, inclination angle, and direction angle are −0.0340 μm, −0.4853″, and −0.0962°, respectively. 

With the identification results of assemble errors and parameters, the electrode gain of every capacitor could also be identified according Equation (56); a proper feedforward gain controller could be settled to compensate the difference of each electrode. In the meanwhile, a Automatic Gain Control Algorithm could be involved in the controller above, and the electrostatic control force applied on resonator and the detection signal on electrode could be both accurate in a certain level, and the accuracy of HRG could be maintained or even improved.

The sensing sensitivity of a sensor is the ratio of output change to input change. For the electrode of a HRG, the sensing sensitivity is the ratio of capacitance change to the vibration displacement change of resonator lip. Let $K_{S}(i)$, ${\hat{K}}_{S}(i)$ be the theoretical sensing sensitivity and identified sensing sensitivity of the *i*-th electrode, respectively. According to Equation (56), the theoretical sensing sensitivity for an eight-electrode HRG will be

(72) $$\{\begin{array}{l} K_{S}(1)=-K_{F}/{\left[d_{a}-\phi R\cos\theta_{\phi }\right]}^{2}\\ K_{S}(2)=-K_{F}/{\left[d_{a}-\phi R\cos\left(\theta_{\phi }-\pi /4\right)\right]}^{2}\\ K_{S}(3)=K_{F}/{\left[d_{a}-\phi R\sin\theta_{\phi }\right]}^{2}\\ K_{S}(4)=K_{F}/{\left[d_{a}-\phi R\sin\left(\theta_{\phi }-\pi /4\right)\right]}^{2}\\ K_{S}(5)=-K_{F}/{\left[d_{a}+\phi R\cos\theta_{\phi }\right]}^{2}\\ K_{S}(6)=-K_{F}/{\left[d_{a}+\phi R\cos\left(\theta_{\phi }-\pi /4\right)\right]}^{2}\\ K_{S}(7)=K_{F}/{\left[d_{a}+\phi R\sin\theta_{\phi }\right]}^{2}\\ K_{S}(8)=K_{F}/{\left[d_{a}+\phi R\sin\left(\theta_{\phi }-\pi /4\right)\right]}^{2} \end{array}$$

To eliminate the difference of sensitivity between the electrodes, feedforward compensation could be involved, and the feedforward compensation gain, for instance, could be simply expressed as

(73) $$K_{C}(i)=8{\hat{K}}_{S}(i)/\sum_{i=1}^{8}{\hat{K}}_{S}(i)$$

Subsequently, the detection signal used for demodulation will be the product of feedforward compensation gain and the signal from electrodes, and the excitation signal that will excite the resonator into vibration state after amplified will be the product of feedforward compensation gain and the signal from controller output.

**Figure 14:**
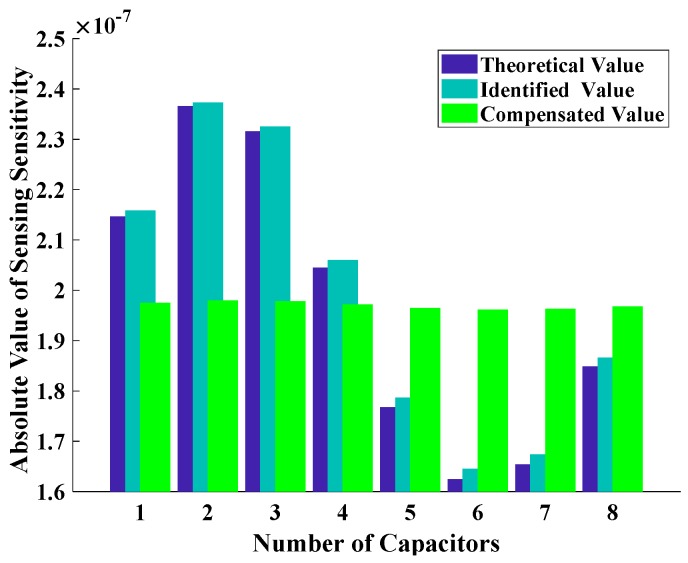
Absolute Value of Sensitivity of Each Capacitor for Eight-Electrode Type HRG.

Suppose the assemble errors and parameters (average gap $d_{a}$, inclination angle ϕ, and direction angle $\theta_{\phi }$) match the condition in this subsection, in particular, to 15 μm, 30″, and 60°, respectively. Subsequently, the absolute value of theoretical sensing sensitivities, identified sensing sensitivities as well as compensated sensing sensitivities for all eight electrodes could be obtained by simulation, and shown in Figure 14.

As shown in the Figure 14, the theoretical sensing sensitivity of each electrode before compensation is different, the difference between maximum and minimum sensing sensitivity takes up 45.61% to the minimum sensing sensitivity. However, after compensation, the proportion will be reduced to 0.94%, which is a remarkable improvement in controlling or detecting the standing wave on resonator.

Besides, the assemble technology of hemispherical shell resonator and flat electrode could be evaluated by estimating the assemble errors in a number of production batches for a mass production situation. To meet the demand of mass production, the assemble procedure should ensure the consistency of assemble errors to avoid deploying complex calibration work for each gyroscope and simplify the gyroscope production process to make an improvement of product reliability level. From this point of view, estimating the assemble errors could facilitate the improvement of HRG manufacturing technology.

## 5. Conclusions

A synthesis model of a flat-electrode hemispherical resonator gyro contained several error and input sources, such as the angle between inherent axes and forcer-pickoff axes, inertial forces, electrostatic control forces, as well as assemble errors, is established by theoretical deduction, part of the synthesis model is verified by simulations, and the assemble errors are identified by the proposed method. 

Firstly, the model of an ideal hemispherical shell resonator with perfect dimension and material parameters are established with the Lagrangian Mechanics Principle and the Elastic Shell Theory, and by introducing the angle error of axes and external forces in an equivalent mechanical model, the motion equations of unideal hemispherical shell resonator are deduced. Not only the error sources that are presented in the classical generalized CVG model are involved here, but also the acquisition method of coefficients is proposed to cover the shortage of the generalized CVG model when it is applied in the analysis of HRG. The coefficients of motion equations are verified to be reliable by comparing with finite element simulation and reported experimental data.

Secondly, based on the model of flat-electrode capacitor, the excitation and detection models with assemble errors and parameters are established, and they are simplified as the linear function form in some situations. The assemble errors and parameters contain inclination angle, direction angle, and average gap. The introduced various parameters could very largely improve the scalability of proposed models. The models convert the form of resonator input from forces to voltage loaded on exciting electrode, and the form of resonator output from displacements to voltage on detecting electrodes. The method could facilitate the application of HRG model during the fabricating procedure of gyroscope, and make it easier to adjust the performance parameters of the gyroscope.

Finally, an identification method of assemble errors and parameters is proposed. The average gap is identified with the average capacitance of all excitation and detection capacitors, and the fitting approximate static capacitor model identifies the inclination angle and direction angle. With the method, the error parameters in excitation and detection model could be finally determined, and the estimation errors could be obtained by simulations. By identifying the assemble errors and parameters, a proper feedforward gain controller could be settled to compensate the difference of electrode gain, the performance of HRG could be maintained or even improved, and the HRG manufacturing technology could be evaluated, which will be beneficial to enhance the ability of HRG mass production.

According to the synthesis model and identification method, a firm and tight connection between the real HRG and theoretical model is established, and a mathematical model with voltage input across exciting electrode and voltage output across the detecting electrode is accomplished. With these results, a fully functional simulation model will be established in the future, and the control and detection methods of a standing wave of HRG could be studied.

## Figures and Tables

**Table 1:** Material and dimension parameters of hemispherical shell resonator.

Parameter	Value	Parameter	Value
Density *ρ*	2.2 × 10^3^ kg/m^3^	Inner radius of hemispherical shell *R*_1_	9.65 × 10^−3^ m
Yong’s Modulus *E*	7.67 × 10^10^ Pa	Outer radius of hemispherical shell *R*_2_	10.35 × 10^−3^ m
Poisson’s Ratio *μ*	0.17	Inner corner radius *r*_1_	2.75 × 10^−3^ m
Diameter of stem *D*	2.2 × 10^−3^ m	Outer corner radius *r*_2_	0.5 × 10^−3^ m

**Table 2:** Static capacitor model simulation parameters.

Parameter	Value	Parameter	Value
*R*_1_	9.65 × 10^−3^ m	*λ*_1_	1.8782 × 10^−12^ F
*R*_2_	10.35 × 10^−3^ m	*λ*_2_	0.0799
*d_a_*	10 × 10^−6^ m	*λ*_3_	2.9856 × 10^4^
*d_e_*	0.4 × 10^−3^ m	*λ*_4_	4.3715 × 10^−12^ F
*φ*	0″ to 100″, interval 10″	*θ* *_φ_*	0° to 345°, interval 15°
